# The Role of Gut Microbiota in Neuropsychiatric Diseases – Creation of An Atlas-Based on Quantified Evidence

**DOI:** 10.3389/fcimb.2022.831666

**Published:** 2022-03-14

**Authors:** Bruno Bonnechère, Najaf Amin, Cornelia van Duijn

**Affiliations:** ^1^ REVAL Rehabilitation Research Center, Faculty of Rehabilitation Sciences, Hasselt University, Diepenbeek, Belgium; ^2^ Nuffield Department of Population Health, University of Oxford, Oxford, United Kingdom

**Keywords:** gut microbiota, psychiatric diseases, autism spectrum disorders, depression, schizophrenia

## Abstract

There is a growing body of evidence highlighting the significant role of gut microbiota in various pathologies. We performed a systematic review to review the different microbiota involved in neuropsychiatric diseases. 50 studies (23 studies for autism spectrum disorders, 18 for major depression, and 9 for schizophrenia), representing 2,137 patients and 2,844 controls. Concerning the microbiota, the genera *Prevotella, Clostridium, Bacteroides, Bifidobacterium, Ruminococcus, Megamonas, and Faecalbacterium* were the ones detected with the most frequent variation of their relatives abundance. We also assess the overlap between the different pathologies. This study provides new insights into the complex relationship between the brain and the gut and the implications in neuropsychiatric pathologies. The identification of unique signatures in neuropsychiatric diseases suggests new possibilities in targeted anti or probiotic treatment.

## Introduction

The past decade has seen increasing interest in the microbiota-gut-brain axis, the bidirectional communication between the brain and the gut (Stilling et al., 2014). The microbiota-gut-brain interaction may play a role in the pathogenesis of various central nervous disorders (Wang and Kasper, 2014) and is, probably, one of the most promising new areas of research (Heiss and Olofsson, 2019; Sasmita, 2019; Seguella et al., 2019) from an epidemiologic and clinical perspective. Recent developments in high throughput DNA sequencing have made it possible to study the microbiota cost-effectively. Changes in the diversity and composition of the gut microbiota have been reported for a wide range of brain disorders and different potential mechanisms of action have been reviewed. For long, the gut microbiota has been linked to the hypothalamic-pituitary-adrenal axis and mood disorders such as bipolar disorders and major depression disorders (MDD) (Cheung et al., 2019; Kelly et al., 2019; Peirce and Alviña, 2019). Also, Autism Spectrum Disease (ASD) is one the diseases that have been studied in depth (Saurman et al., 2020) and there is increasing interest in the gut-brain axis in schizophrenia research (Golofast and Vales, 2020). There is increasing interest in the role of the gut microbiota in major depression, the most common psychiatric disorder. Overactivity of the hypothalamic-pituitary-adrenal (HPA) axis in major depression, a link between HPA axis activity and cognitive performance, and a possible involvement of HPA axis genetic variation in cognition have all been discovered in neuroendocrine investigations (Keller et al., 2017). Changes in gut microbiota composition could increase gut barrier permeability, activate systemic inflammation and immune responses, regulate monoamine neurotransmitter release and efficacy, change the activity and function of the HPA axis, and change the abundance of brain-derived neurotrophic factor, all of which could lead to depression (Du et al., 2020).

Despite the growing body of evidence, there is a lack of quantitative evidence about the different microbiota involved in psychiatric diseases. However, many questions remain to be answered. Are gut microbiota are implicated consistently to a specific disorder across studies? Are the associations unique to the disease or are the associations also seen in other psychiatric diseases? To answer these questions we have reviewed the direction of the relationship between microbiota and disease across various neurologic and neuropsychiatric diseases.

## Methods

### Search Strategy

**Figure 1:**
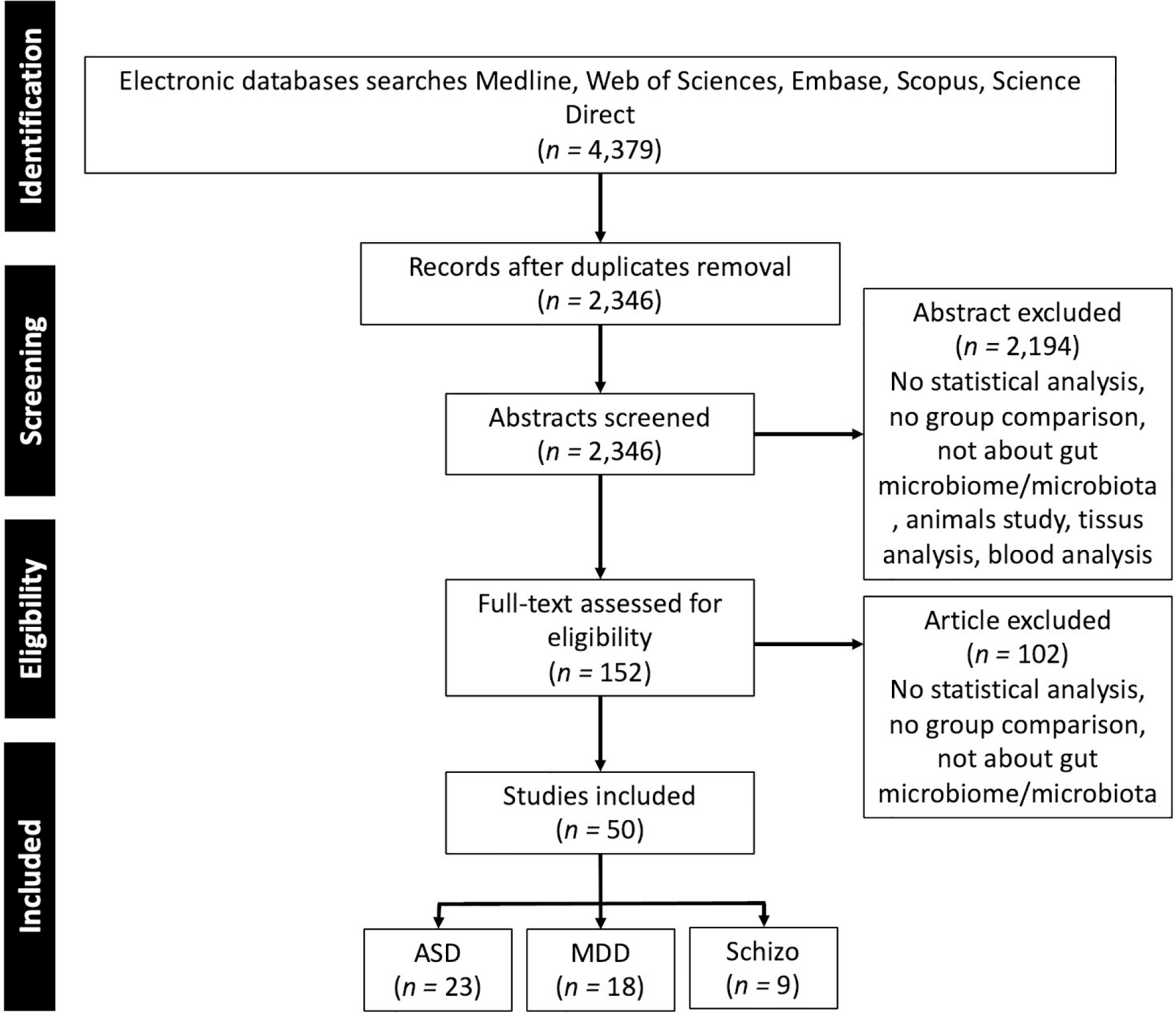
PRISMA flow diagram of the studies selection. ASD, Autism Spectrum Disorder; MDD, Major Depressive Disorder; Schizo., Schizophrenia.

To identify the microbiota associated with neurological and neuropsychiatric disorders in the human population we performed a literature review that included articles published prior to 1^st^ January 2021 with a combination of term “gut”, “microbiota”, “microbiome”, “stool”, “fecal” and the different pathologies “Depression”, “MDD”, “Autism”, “Autism spectrum disorders”, “Schizophrenia”. Additional relevant articles were sought through a manual bibliography search. Inclusion criteria were: human research, focus on depression, ASD, schizophrenia that makes a comparison between patients and healthy controls, focus on gut microbiota quantified from stool samples, articles published in English in peer-reviewed journals. A flow diagram of study selection is presented in **Figure 1**.

### Data Extraction

From the included studies the following parameters were extracted: the country where the experiments have been carried out, general characteristics of the patients and control (e.g., age, BMI, sex ratio), microbiota analysis method. We then listed the different taxon of which relative abundance has been found statistically significantly different between patients and healthy controls and indicated the direction: relative abundance increased or decreased in the various pathologies.

### Statistical Analysis

We determined whether findings were consistent across studies of a single disorder based on statistical significance and the direction of association for the studies that showed significant results. If two studies were found to show the same direction of the relationship (e.g., microbiota Taxus was found more often in patients in both studies), findings were classified as consistent. As most studies were small, one expects heterogeneity across studies due to random fluctuation. We therefore also considered findings for which the consistency between was more than 50% as consistent. We present the phylogenetic trees of the microbiota that have been successfully found in the literature for the different pathologies using the GraPhIAn package in Python (Asnicar et al., 2015). General information about the included studies and baseline data on patients were summarized in graphics. To compare the different microbiota involved in the studies’ pathologies we used Venn diagrams using the VennDiagram package in R (Chen and Boutros, 2011).

## Results

**Figure 2:**
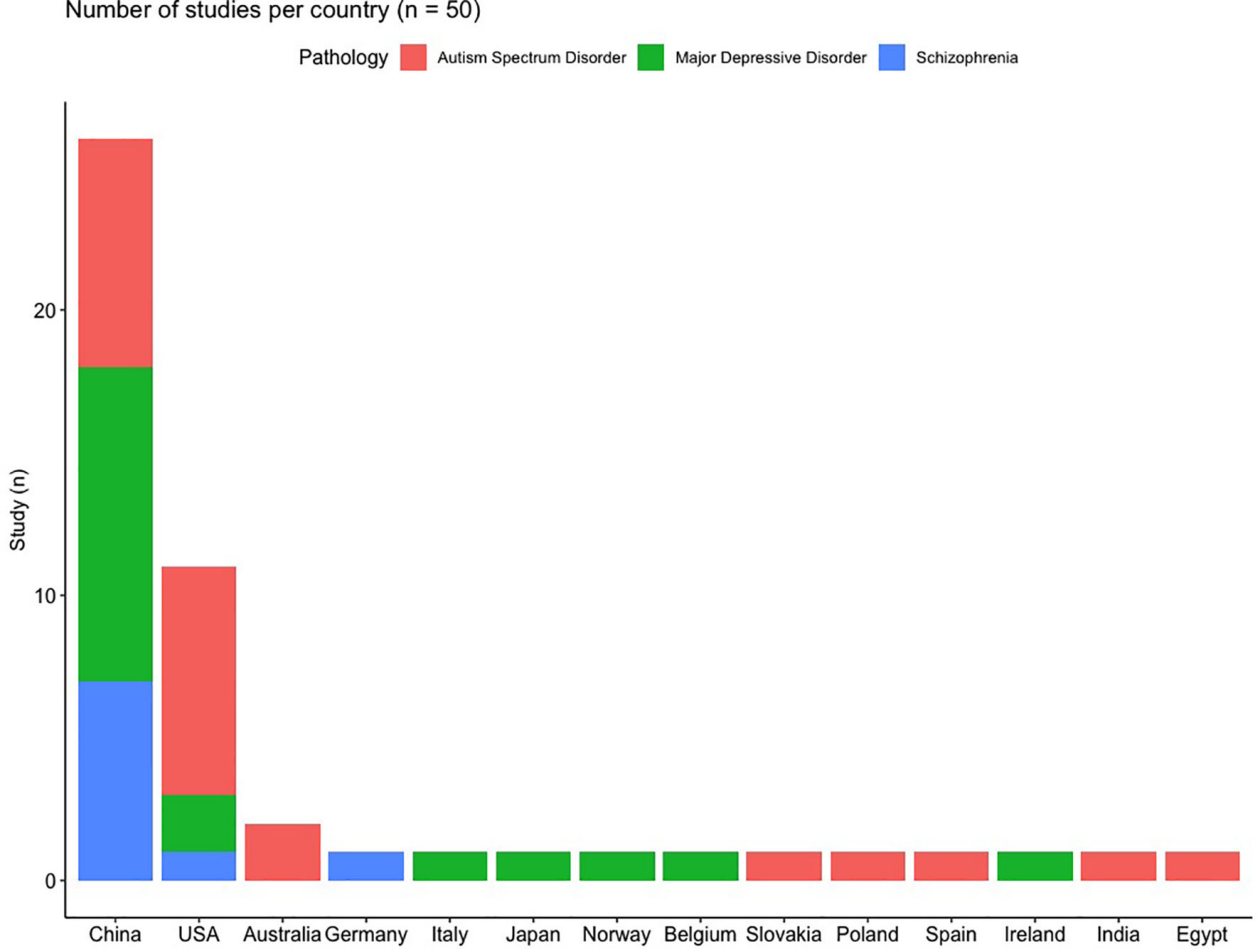
Distribution of the included studies according to the countries and studied pathologies.

In total, 50 studies were included in this review, representing 2,137 patients and 2,844 controls, the repartition of the patients within the different pathologies is presented in **Supplementary Figure 1**. ASD is the most frequently studied disorder (**Figure 1** and **Supplementary Figure 1**), comprising 46% of all patients and 44% of all studies. As the gut microbiota is known to depend on the environment (Anwar et al., 2021) and diet (Zmora et al., 2019), which differ across societies and cultures and thus across studies, the distribution of the studies per country is presented in **Figure 2**. It is of note that most of the studies were conducted in China (*n =* 26, 52%) and in the USA (n = 11, 22%), only 8 (16%) of the included studies were done in Europe.

The characteristics for patients with ASD are presented in **Supplementary Table 1**, for depression in **Supplementary Table 2**, and for schizophrenia in **Supplementary Table 3**.

**Figure 3:**
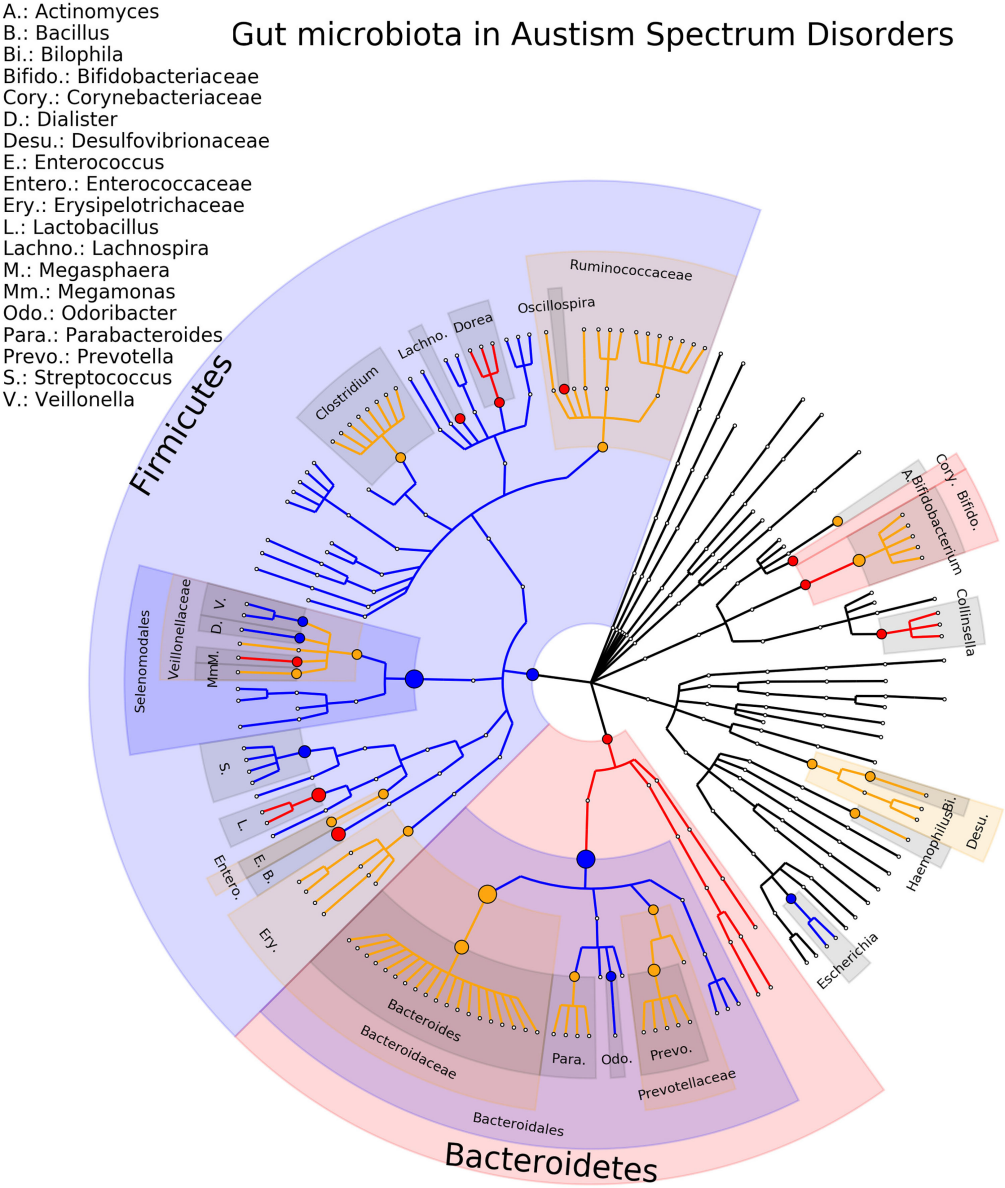
Phylogenetic distributions of the microbiota involved in Autism Spectrum Disorder. Orange indicates inconsistent results, blue for a decrease in relative abundance and red increased in relative abundance.

The most studied neuropsychiatric disorder is ASD with twenty-three studies included in the review, involving a total of 932 patients and 792 healthy controls (Adams et al., 2011; Wang et al., 2011; Gondalia et al., 2012; Kang et al., 2013; Son et al., 2015; Tomova et al., 2015; Luna et al., 2016; Strati et al., 2017; Iovene et al., 2017; Kushak et al., 2017; Kang et al., 2018; Pulikkan et al., 2018; Zhang et al., 2018; Rose et al., 2018; Liu et al., 2019; Zhai et al., 2019; Ma et al., 2019; Plaza-Díaz et al., 2019; Sun et al., 2019; Chen et al., 2020; Ding et al., 2020; Zou et al., 2020; Ahmed et al., 2020). At the genus level, there are 64 gut microbiota-disease associations reported. Of these, 12 (19%) associations are consistent (*Bacillus, Collinsella, Dialister, Dorea, Escherichia/Shigella, Lachnospira, Lactobacillus, Megaspharea, Odoribacter, Oscillospira, Streptococcus, Veillonella*) and for 4 there are multiple studies in the same direction (*Escherichia/Shigella, Lachnospira, Lactobacillus, Streptococcus*). For 14 (25%) gut microbiota, an opposite direction of association is reported (*Actinomyces, Akkermansia, Bacteroides, Bifidobacterium, Bilophila, Clostridium*, *Erysipelatoclostridium, Faecalbacterium, Haemophilus, Lachnoclostridium, Megamonas, Parabacteroides, Prevotella, Ruminococcus*). The phylogenetic tree representing the results are presented in **Figure 3**, the complete results of the individual studies are presented in **Supplementary Table 4**. Of note is that some studies did not find any statistical differences between ASD patients and controls (Gondalia et al., 2012; Son et al., 2015).

**Figure 4:**
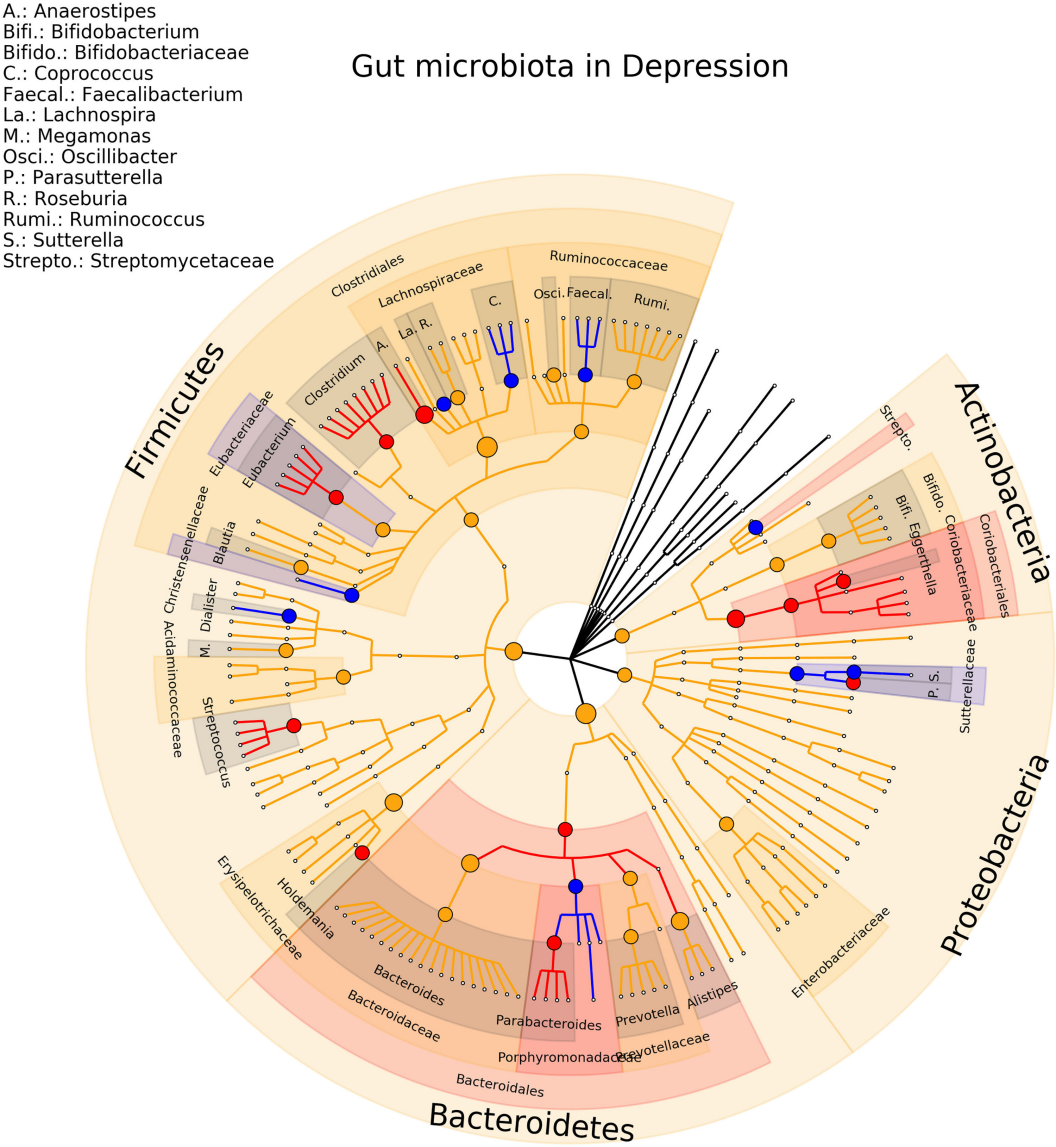
Phylogenetic distributions of the microbiota involved in Major Depressive Disorder. Orange indicates inconsistent results, blue for a decrease in relative abundance and red increased in relative abundance.

Eighteen studies on MDD were included in the review, involving a total of 801 depressed patients and 1,634 healthy controls (Naseribafrouei et al., 2014; Jiang et al., 2015; Aizawa et al., 2016; Zheng et al., 2016; Kelly et al., 2016; Liu et al., 2016; Lin et al., 2017; Chen et al., 2018; Chen et al., 2018; Valles-Colomer et al., 2019; Lai et al., 2019; Chung et al., 2019; Rong et al., 2019; Mason et al., 2020; Fontana et al., 2020; Zheng et al., 2020; Liu et al., 2020; Chen et al., 2020). At the genus levels, 21 associations were detected in only one study, 12 (27%) association were consistent in term of direction and significance (*Anaerostipes, Coprococcus, Dialister, Eggerthella, Eubacterium, Faecalbacterium, Holdemania, Lachnospira, Parabacteroides, Parasutterella, Streptococcus, Sutterella*). For the other associations, *Bacteroides* concentration was found increased in four studies and decreased in two studies, *Blautia* concentration was found increased in 4 studies while the relative abundance was found decreased in two studies, for *Clostridium* 5 studies found an increase of the relative abundance and only one study found a decreased concentration, for *Oscillibacter* 3 studies found an increase and only one a decrease of the relative abundance, for *Bifidobacterium* and *Prevotella* 3 studies found a decrease and 2 and increase of the relative abundance finally three associations (*Alistipes, Megamonas, Ruminococcus*) are found in the same direction in 2 studies and on the opposite direction in 1 study. The phylogenetic tree representing the results are presented in **Figure 4**. Complete results of the included studies are presented in **Supplementary Table 5**.

**Figure 5:**
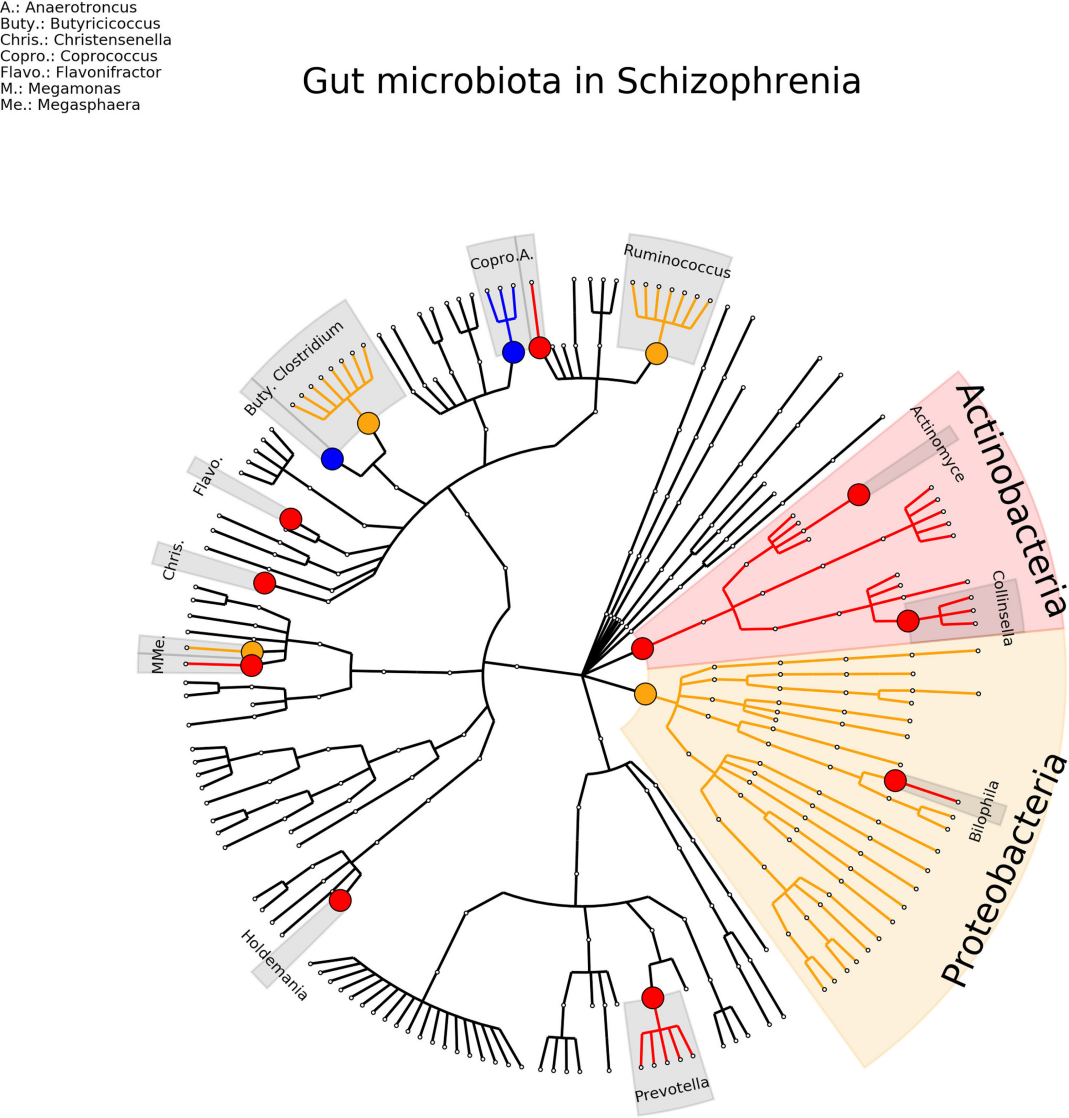
Phylogenetic distributions of the microbiota involved in Schizophrenia. Orange indicates inconsistent results, blue for a decrease in relative abundance and red increased in relative abundance.

For schizophrenia, nine studies were included, involving a total of 404 patients and 418 healthy controls (Schwarz et al., 2018; Shen et al., 2018; Nguyen et al., 2019; Xu et al., 2019; Li et al., 2020; Ma et al., 2020; Pan et al., 2020; Xu et al., 2020; Zhang et al., 2020). 11 (16%) genera were found consistent in term of direction and significance (*Actinomyces, Anaerococcus, Bilophila, Butyricicoccus, Christensenella, Collinsella, Coprococcus, Faecalbacterium, Flavonifractor, Holdemania, Prevotella*) and 5 (7%) were inconsistent in term of direction *(Blautia, Clostridium, Eisenbergiella, Megasphaera, Ruminococcus*) (see **Figure 5** and **Supplementary Table 6**).

The picture emerging is that for ASD association of 6 genera is replicated in 3 studies or more. For depression 12 associations were consistent but for 10 findings were in opposite direction. Finally, for schizophrenia, only 1 association was found consistent in 3 studies and 11 in two studies.

**Figure 6:**
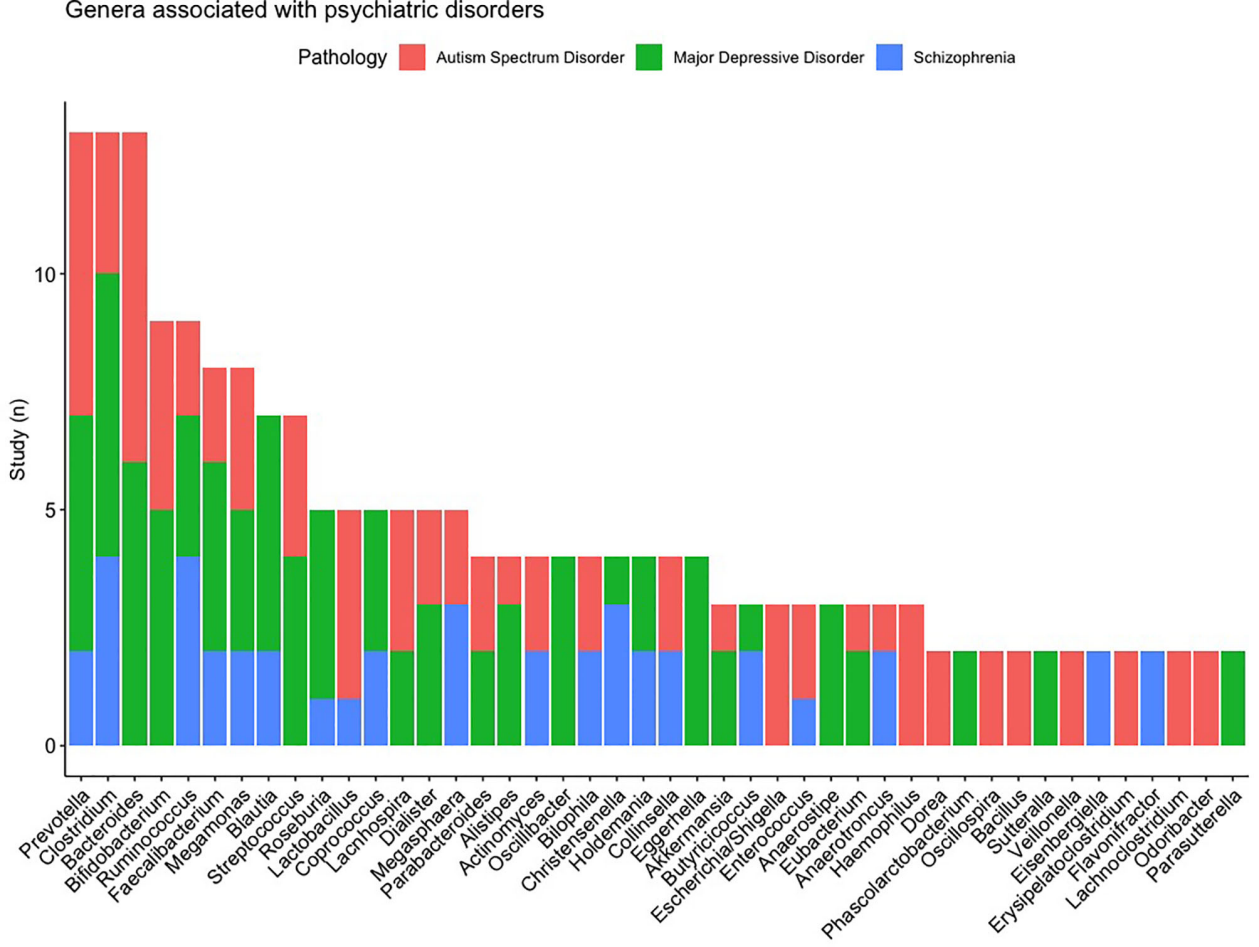
List of the microbiota the most frequently found and their distributions according to pathologies.

First, we started from the microbiota and determined whether the association was reported to different pathologies. **Figure 6** shows the microbiota which are associated with multiple disorders and the number of studies that found the association of the genus to a disease. *Prevotella* and *Clostridium* are associated with multiple psychiatric disorders (the 3 studied disorders), the genus of *Bacteroides* is associated to MDD and ASD. It is interesting to note that the directions are inconsistent across the pathologies and studies.

**Figure 7:**
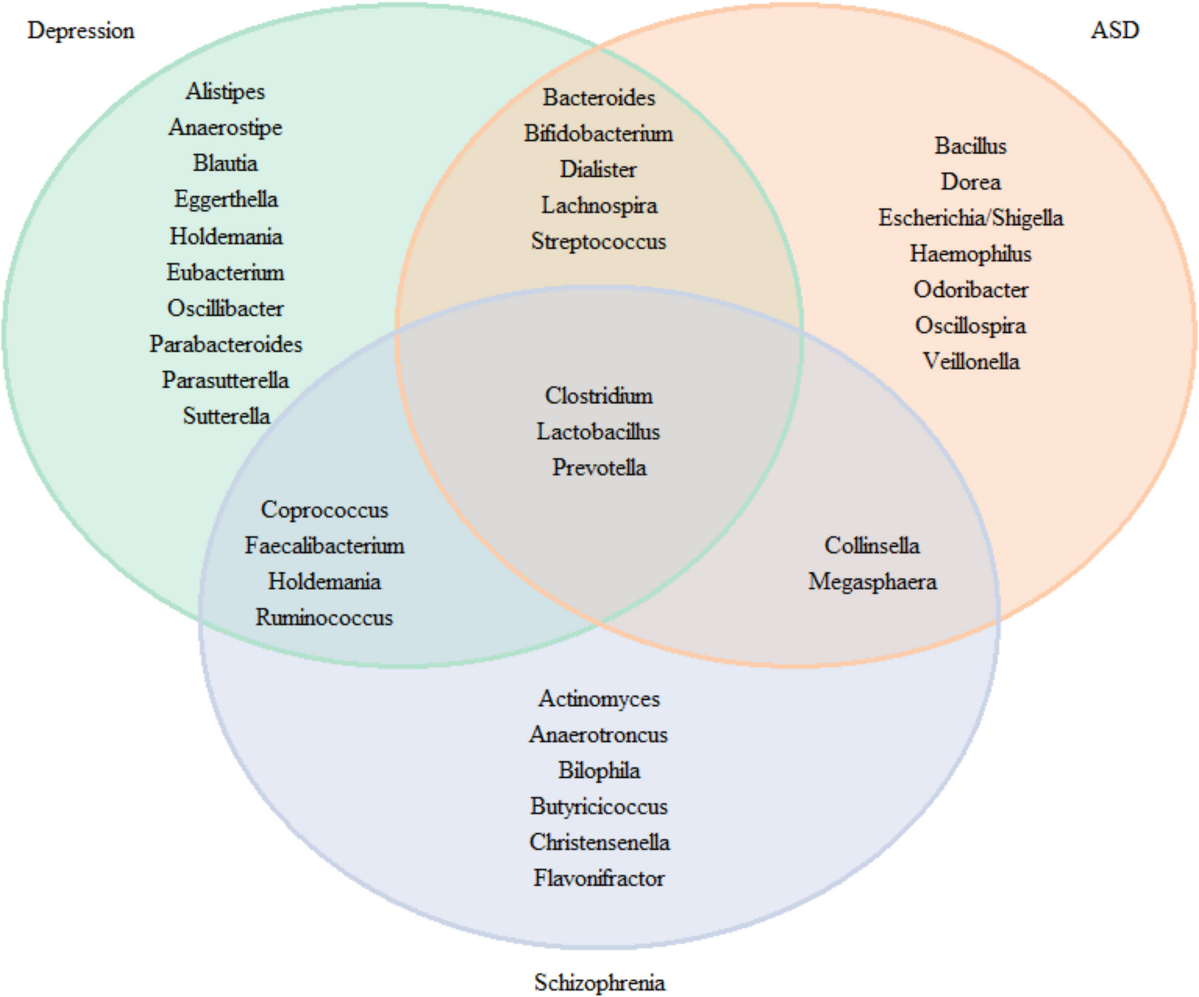
Venn diagram for the genus consistently found in the literature for neuropsychiatric diseases.

For research where inconsistency was discovered across trials, we define consistency as more than 50% of studies consistently finding an association in the same direction. In **Figure 7**, we plotted Venn diagrams summarizing association to the neuropsychiatric disorders and check for overlap microbiota associated. We observed an overlap of the three pathologies for the *Clostridium*, *Lactobacillus* and *Prevotella.* Five genera were found in MDD and ASD (*Bacteroides, Bifidobacterium, Dialister, Lachnospira* and *Steptococcus*), 4 between MDD and schizophrenia (*Coprococcus, Faecalbacterium, Holdemania*, and *Ruminococcus*) and 2 between ASD and schizophrenia (*Collinsella* and *Megasphaera*).

**Figure 8:**
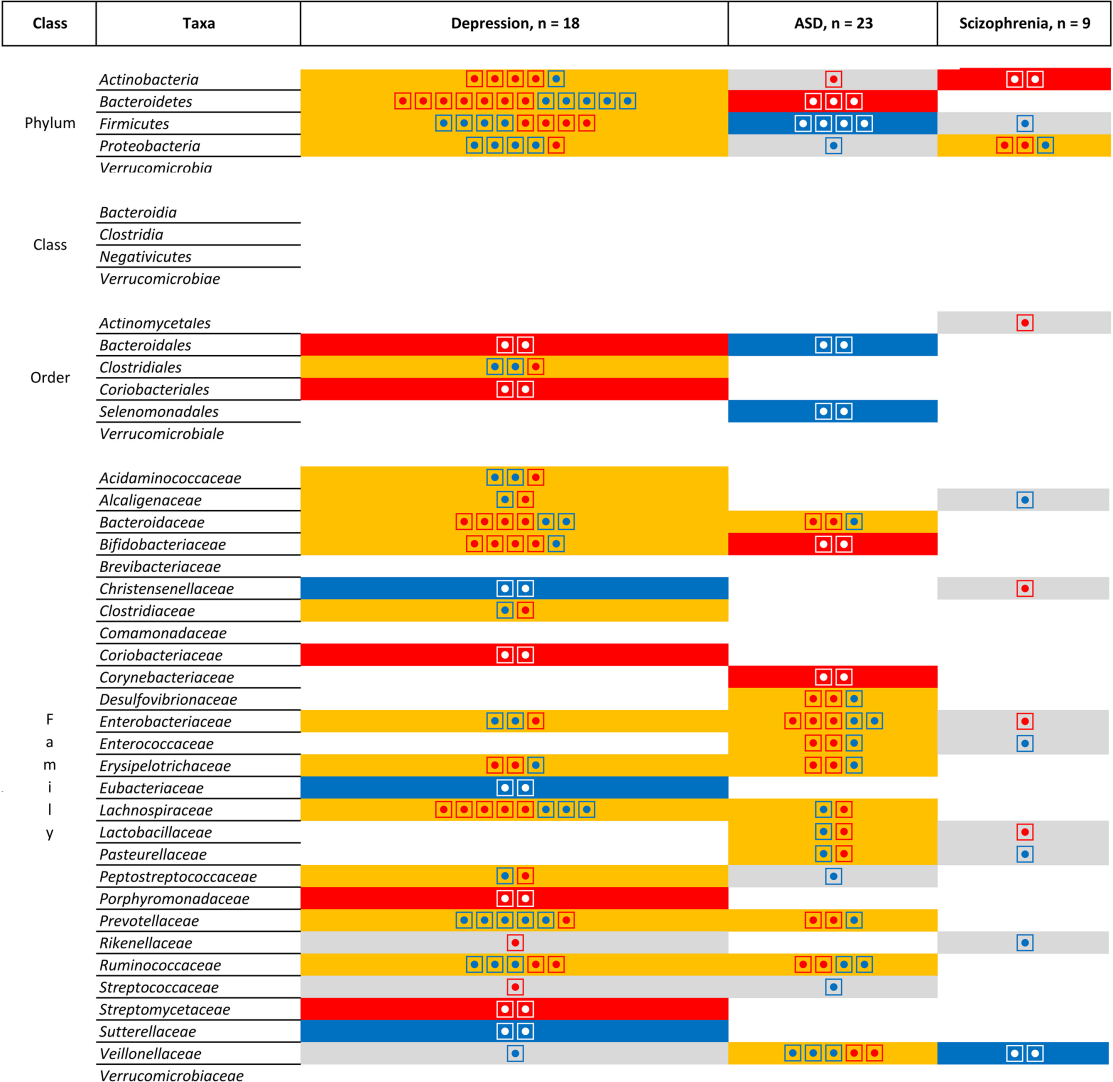
List of the microbiota that have been identified in at least two studies for the same pathologies at the Phylum, Class, Order and Family levels. Grey is use for one study, orange indicates inconsistent results, blue for a decrease in relative abundance and red increased in relative abundance. Each individual circle indicates a study that identify the microbiota.

**Figure 9:**
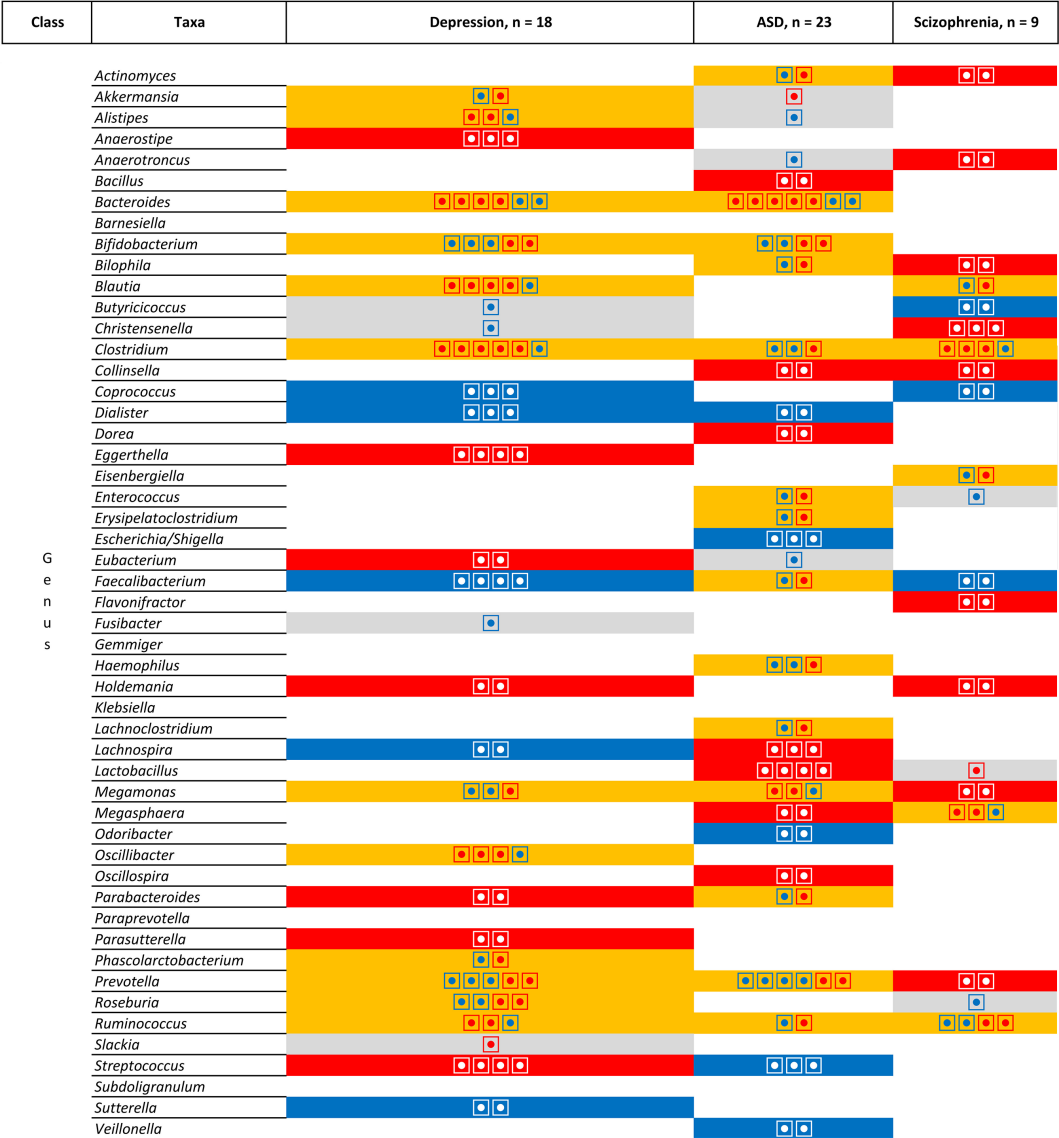
List of the microbiota that have been identified in at least two studies for the same pathologies at the genus level. Grey colour is used for one study, orange colour indicates inconsistent results, blue colour for consistent results showing a decrease in relative abundance in patients compared to patients and red colour for consistent results showing an increased in relative abundance in patients compared to patients. Each individual circle indicates a study that identify the microbiota.

However, **Figure 7** does not take into account the direction of association. When comparing the direction of the association [**Figure 8** (phylum, classer, order and family levels) and **Figure 9** (genus level)], for MDD and ASD we found an increase in the relative abundance of *Bacteroides* and a decrease in the abundance of *Dialister* and *Prevotella*; opposite direction are found for *Clostridium* (increase in depression), *Lachnospira* (decrease in depression), *Lactobacillus* (decrease in depression), and *Streptococcus* (increase in depression). When comparing ASD and schizophrenia *Collinsella*, *Megamonas*, *Megasphaera* are found in the same direction (increase of relative abundance), while *Clostridium* and *Prevotella* are found in the opposite direction (decrease in ASD). Finally for depression and schizophrenia *Clostridium*, *Collinsella*, *Holdemania* are increased, *Coprococcus* and *Faecalbacterium* are decreased and opposite direction is found for *Megamonas* (decrease in depression) and *Prevotella* (decrease in depression).

## Discussion

By far the most studied pathologies is ASD as well as the highest level of consistency as highlighted by the phylogenetic trees (**Figure 3**). Several studies (Luna et al., 2016; Finegold et al., 2017) compared patients with functional gastrointestinal disorders (FGID) and controls without FGID making the comparison of gut microbiota difficult to interpret since the two factors play a role in the composition of microbiota. Only one study compared ASD children with and without FGID with controls with and without FGID (Rose et al., 2018). Authors found differences at the Family level: Bacteroidaceae, Lachnospiraceae, Prevotellaceae and Ruminocaccaceae were more abundant in ASD with FGID compared to control with FGID, interestingly the same differences were not detected between patients and controls without FGID (Rose et al., 2018). For major depressive disorders although the number of included studies is high (n = 18) we did not find such consistency between the studies as for ASD (**Figures 3**, **4**). However the field is growing and the evidences starting to emerge. Interestingly it has been demonstrated that women with post-partum depression also presented significant modifications of the microbiota (Zhou et al., 2020), which seems to indicate that the changes are occurring quite quick and are not associated to the chronicity of the MDD. The age of the patients also influences the modification seen in depression (Chen et al., 2020). Finally a study of twins with depression shows that the affected twins had a lower diversity and an absence of a specific operational taxonomical unit in comparison with healthy control (Vinberg et al., 2019). This leads us to question the impact and the interactions between the gene and the environment on the observed modifications.

There are several possible explanations about the mechanisms of action driving the relationship between gut microbiota and the different diseases. A recent review underlines the relationship among inflammation, pain, microbiota and the different lipids, focusing on the possible involvement of N-acylethanolamine family and short-chain fatty acids in the gut-brain axis and their role in the central nervous system diseases (Russo et al., 2018). The lymphatic system could be the mediator of this communication between the gut microbiota and the brain (Tsunoda, 2017).

In this review, we summarized the current evidence of the modification of the gut microbiota in various pathologies. We limited our analysis to the main neuropsychiatric diseases but gut microbiota composition is also modified in other neurological disorders such as Rett syndrome (Strati et al., 2016) and in neurocritically ill patients (Xu et al., 2019). However, their roles are not limited to neurological or neuropsychiatric diseases, they are also involved in hypertension (Li et al., 2017; Yan et al., 2017), cirrhosis (Bajaj et al., 2012; Bajaj et al., 2014) or primary hepatocellular carcinoma (Ni et al., 2019), diabetes (Chen et al., 2019), autoimmune diseases (Chen et al., 2017), Systemic Lupus Erythematosus (Luo et al., 2018), systemic immunity in allergic disease (Ipci et al., 2017), Behcet’s disease (Ye et al., 2018), systemic sclerosis (Patrone et al., 2017), rheumatoid arthritis (Liu et al., 2013) and could also potentially influence vitamin D production (Zuo et al., 2019) and there are correlation between some gut microbiota and personality in adults (Kim et al., 2018).

An essential part of the research in this field is the development of treatments to modify the microbiota. For example, intermittent fasting led to increased gut bacteria richness, enrichment of the Lactobacillaceae, Bacteroidaceae, and Prevotellaceae families and enhanced antioxidative microbial metabolic pathways (Cignarella et al., 2018). Changes in microbiota composition and clinical improvement have also been observed after specific diet (Libbey et al., 2018; Rice et al., 2019). Other promising results and modification of the gut microbiota have been obtained with fecal transplantation in animals and human studies (Evrensel and Ceylan, 2016; Sun et al., 2018). So far the most promising results of clinical improvement after modification of the gut microbiota have been obtained in patients with epilepsy after ketogenic diet (Zhang et al., 2018; Fan et al., 2019; Lindefeldt et al., 2019). The researchers showed that there was an overall decrease in the mean species diversity after treatment and importantly, a difference between the variation of species between responders and non-responders. Further analysis of species composition before and after treatment showed a significant increase in *Bacteroides* and a decrease in *Firmicutes* and *Actinobacteria*. When comparing responders and non-responders, *Clostridiales*, *Clostridia*, *Ruminococcaeceae*, *Lachnospiraceaea*, *Alistipes* and *Tikenellacase* were significantly increased in non-responders (Spinelli and Blackford, 2018).

Another main field of research is to determine if modification of the gut microbiota can decrease the risk of developing disease or improve the health of the patients: in Alzheimer’s disease (Mancuso and Santangelo, 2018), Parkinson’s disease (Sun and Shen, 2018), multiple sclerosis (Mirza and Mao-Draayer, 2017), amyotrophic lateral sclerosis (Zhang et al., 2017), and epilepsy (De Caro et al., 2019).

There are a few limitations in this review. The first one concerns the methodology used in the different studies: all studies used 16sRNA analysis but in some studies, corrections were applied for medication or constipation, two factors known to modify the composition of gut microbiota, while in most of the other studies these factors were not taken into consideration. Another technical point is that some authors applied corrections for multiple testing, some not and the techniques used are also different [e.g. Bonferroni’s correction for multiple testing or false discovery rate (Brenner et al., 2018)]; the results are different adjusting or not for multiple analyses [see for example study of Kang et al. presenting both results (Kang et al., 2018)]. Other studies used linear discriminant analysis (LDA) (usually LDA > 2.0) to differentiate patients and control (Liu et al., 2019). For all of the above limitations, we were not able to perform a meta-analysis which could provide a clearer vision of the direction and intensity of the modifications observed in the different diseases.

Because of a lack of existing data, we were not able to assess sex difference while it seems that, at least for some pathologies, there are differences between male and female (Chen et al., 2018; Holingue et al., 2020; Jaggar et al., 2020), as well as the ethnicities (Ventura et al., 2019). We also limit our analysis to genetic analysis of gut microbiota composition. Recent research also evaluate immune-inflammation (Carissimi et al., 2019) and gut glutamate metabolism (Wang et al., 2019) to differentiate patients and healthy controls.

The main limitation of the current studies are the populations investigated. We have seen that a majority of the studies have been done in China and in USA. This high homogeneity made the generalization and the translation of the results for these pathologies more limited since it has been shown that genetics (Simões et al., 2013; Matsuki et al., 2016), socioeconomic status (Bowyer et al., 2019), host and environmental factors (Anwar et al., 2021) and diet influence the composition of the microbiota serve a significant role in shaping the gut microbiota population (Bibbò et al., 2016; Chassaing et al., 2017; Proctor et al., 2017; Sandhu et al., 2017).

Of note is that we limited our analysis to binary responses between patients and controls but it is interesting to analyse the microbiota concentration as continuous variables since the severity of some diseases is correlated with the relative abundance of various microbiota. It has, for example, been shown that a greater severity of depressive symptoms was correlated with a greater abundance of genus *Bacteroides* (*r* = 0.70; *p* = 0.0002), while increased negative symptoms were associated with decreased abundance of family Ruminococcaceae (*r* = −0.74; *p* = 0.0002). Overall self-reported mental well-being was positively correlated with phylum Verrumicrobia (*r* = 0.63; *p* = 0.002) (Nguyen et al., 2019).

Another limitation is that we only included results of the studies using DNA sequencing of the fecal stool while it may also be interesting to study the composition of microbiota from different samples (Park et al., 2017). Depending on the diseases, other biota can be analysed and be a good indicator of the severity of the diseases, such as mouth microbiota in schizophrenia (Castro-Nallar et al., 2015; Yolken et al., 2015). Although the analysis of other microbiota may be challenging from the point of view of tissue collection, it has been showed that brain (Branton et al., 2016) – blood (Olde Loohuis et al., 2018) or direct microbiota analysis from the intestinal tissue (Cosorich et al., 2017) in patients multiple sclerosis could provide different type of information.

In the future analysing the changes in microbiota from nose to gut could also be an essential source of knowledge’s to get a better insight into the diseases (Bell et al., 2019). Finally, we limited our analysis at the genus level using 16S sequencing while it is possible to investigate the bacterial species in depth by sequencing the full genome, but only a few studies are currently performing this kind of analysis requiring deep sequencing methods.

## Conclusion

The gut microbiota plays an important role in the development of neuropsychiatric diseases. We summarized the current pieces of evidence about the quantified modifications of the gut microbiota compositions in patients compared to healthy controls. The most remarkable microbiota signature was observed in ASD. For future clinical applications, baseline value and standard reporting template have just been developed that should ease and spread the analysis of gut microbiota in daily practice for the clinicians (King et al., 2019). The analysis of the gut microbiota could therefore be used to refine diagnostic or to assess the severity of MDD (Averina et al., 2020). There is currently an urgent need for standardization of the analysis and report of studies related to gut microbiota modification in order to move this field forward.

## Author Contributions

The study was conceived by BB and CD. NA and CD verified the analytical methods. BB, NA, and CD did the data interpretation. CD supervised the findings of this work. All authors discussed the results and contributed to the final manuscript. All authors contributed to the article and approved the submitted version.

## Conflict of Interest

The authors declare that the research was conducted in the absence of any commercial or financial relationships that could be construed as a potential conflict of interest.

## Publisher’s Note

All claims expressed in this article are solely those of the authors and do not necessarily represent those of their affiliated organizations, or those of the publisher, the editors and the reviewers. Any product that may be evaluated in this article, or claim that may be made by its manufacturer, is not guaranteed or endorsed by the publisher.
