# Supplementary material for: The Role of Gut Microbiota in Neuropsychiatric Diseases – Creation of An Atlas-Based on Quantified Evidence
Source: Front Cell Infect Microbiol. 2022 Mar 14;12:831666. doi: 10.3389/fcimb.2022.831666 (PMC8964285; doi:10.3389/fcimb.2022.831666)
Supplement: Supplementary file 1 [file Table_1.docx]

**Supplementary Table 1**: Characteristics of the included studies on Autism Spectrum Disorders (ASD).

| Study | Country | Participants | Microbiota analysis method |
| --- | --- | --- | --- |
| Adams et al. 2011(Adams et al., 2011) | USA | 58 patients with ASD (6.9 ± 3 years old, BMI unknown, 8 female)  39 control (7.7 ± 4 years old, BMI unknown, 21 female). | 16S rRNA amplicon (V4 regions) sequencing analysis |
| Wang et al. 2011(Wang et al., 2011, 2013) | Australia | 23 patients with ASD (10.1 ± 1 years old years old, BMI unknown, 2 female)  22 typically developing sibling (12.1 ± 1 years old, BMI unknown, 11 female)  9 independent control (10.2 ± 1 years old ,5 female) | Primers and optimized quantitative real-time PCR |
| Gondalia et al. 2012(Gondalia et al., 2012) | Australia | 51 patients with ASD (2 – 12 years old, BMI unknown, 9 female)  53 control (2 - 12 years old, BMI unknown, 34 female). | 16S rRNA amplicon (V3-4 regions) sequencing analysis |
| Kang et al. 2013(Kang et al., 2013) | USA | 20 patients with ASD (6.7 ± 3 years old, BMI unknown, 2 female)  20 control (8.3 ± 4 years old, BMI unknown, 3 female). | 16S rDNA-targeting quantitative real-time PCR (qPCR) with triplicate PCR |
| Son et al. 2015(Son et al., 2015) | USA | 59 patients with ASD (10.3 ± 2 years old, BMI unknown, 7 female)  44 typically developing sibling (10.0 ± 2 years old, BMI unknown, 23 female). | 16S rRNA amplicon (V1-V2 and V3-V4 regions) sequencing analysis |
| Tomova et al. 2015(Tomova et al., 2015) | Slovakia | 10 patients with ASD (2 - 9 years old, BMI unknown, 1 female)  9 typically developing sibling (5 - 17 years old, BMI unknown, 2 female)  10 control (2 – 11 years old, BMI unknown, 2 female) | 16S rRNA amplicon (V4 regions) sequencing analysis |
| Strati et al. 2017(Strati et al., 2017) | Italy | 40 patients with ASD (11.1 ± 7 years old, BMI unknown, 9 female)  40 control (9.2 ± 8 years old, BMI unknown, 12 female). | 16S rRNA amplicon (V3-V5 regions) sequencing analysis |
| Iovene et al. 2017(Iovene et al., 2017) | Italy | 47 patients with ASD (6.0 ± 3 years old, BMI unknown, 7 female)  33 control (7.3 ± 3 years old, BMI unknown, 19 female). | 16S rRNA amplicon (V3-V4 regions) sequencing analysis |
| Luna et al. 2017(Luna et al., 2016, 201) | USA | 14 patients with ASD and functional gastro-insterinal disorders (FGID) (8.5 [4 – 13] years old, BMI unknown, 0 female)  15 control with FGID (10.5 [3 – 18] years old, BMI unknown, 3 female).  6 control without FGID (5.5 [3 – 14] years old, BMI unknown, 0 female) | 16S rRNA amplicon (V1-V3 and V4 regions) sequencing analysis |
| Finegold et al. 2017(Finegold et al., 2017) | USA | 33 patients with ASD and FGID (6.0 ± 3 years old, BMI unknown, 7 female)  13 control without FGID (7.3 ± 3 years old, BMI unknown, 19 female). | 16S rRNA amplicon (V4 regions) sequencing analysis |
| Kushak et al. 2017(Kushak et al., 2017) | USA | 21 patients with ASD (14.4 ± 1 years old, BMI unknown, 2 female)  19 control (16.1 ± 1 years old, BMI unknown, 9 female). | 16S rRNA amplicon (V4 regions) sequencing analysis |
| Kang et al. 2018(Kang et al., 2018) | USA | 23 patients with ASD (10.1 ± 4 years old, BMI unknown, 1 female)  21 control (8.4 ± 3 years old, BMI unknown, 6 female). | 16S rRNA amplicon (V2-V3 regions) pyrosequencing analysis |
| Pulikkan et al. 2018(Pulikkan et al., 2018) | India | 30 patients with ASD (9.3 ± 3 years old, 14.9 ± 2 kg/m², 2 female)  24 family-matched control (8.9 ± 3 years old, 17.5 ± 5 kg/m², 9 female). | 16S rRNA amplicon (V4 regions) sequencing analysis |
| Zhang et al. 2018(Zhang et al., 2018) | China | 35 patients with ASD (4.9 ± 1 years old, BMI unknown, 6 female)  6 control (4.6 ± 1 years old, BMI unknown, 1 female). | 16S rRNA amplicon (V4 regions) pyrosequencing analysis |
| Rose et al. 2018(Rose et al., 2018) | USA | 21 patients with ASD and FGID (6.6 [5 – 9] years old, BMI unknown, 4 female)  29 patients with ASD without FGID (7.7 [5 – 11] years old, BMI unknown, 4 female)  7 control with FGID (5.1 [4 – 6] years old, BMI unknown, 1 female).  34 control with FGID (7.1 [6 – 9] years old, BMI unknown, 2 female). | 16S rRNA amplicon (V3-V4 regions) sequencing analysis |
| Liu et al. 2019(Liu et al., 2019) | China | 30 patients with ASD (4.4 ± 1 years old, BMI unknown, 5 female)  20 control (4.3 ± 1 years old, BMI unknown, 4 female). | 16S rRNA amplicon (V3-V4 regions) sequencing analysis |
| Zhai et al. 2019(Zhai et al., 2019) | China | 78 patients with ASD (4.9 ± 1 years old, BMI unknown, 22 female)  58 control (4.9 ± 1 years old, BMI unknown, 27 female). | 16S rRNA amplicon (V3-V4 regions) sequencing analysis |
| Ma et al. 2019(Ma et al., 2019) | China | 45 patients with ASD (7.3 ± 1 years old, BMI unknown, 6 female)  45 control (4.3 ± 1 years old, BMI unknown, 6 female). | 16S rRNA amplicon (V3-V4 regions) sequencing analysis |
| Plaza-Diaz et al. 2019(Plaza-Díaz et al., 2019) | Spain | 48 patients with ASD (3.8 ± 3 years old, 15.9 ± 1 kg/m², sex unknown)  57 family-matched control (8.9 ± 3 years old, 17.5 ± 5 kg/m², sex unknown). | 16S rRNA amplicon (V3-V4 regions) sequencing analysis |
| Sun et al. 2019(Sun et al., 2019) | China | 9 patients with ASD (7.3 ± 1 years old, BMI unknown, 6 female)  6 control (4.3 ± 1 years old, BMI unknown, 6 female). | 16S rRNA amplicon (V3-V4 regions) sequencing analysis |
| Chen et al. 2020(Chen et al., 2020) | China | 76 patients with ASD (4.0 ± 1 years old, 15.6 ± 1 kg/m², 15 female)  47 control (4.2 ± 1 years old, 16.3 ± 1 kg/m², 6 female). | 16S rRNA amplicon (V3-V4 regions) sequencing analysis |
| Ding et al. 2020(Ding et al., 2020) | China | 77 patients with ASD (38.5 ± 12 months old, BMI unknown, 18 female)  50 control (42.9 ± 14 months old, BMI unknown,11 female). | 16S rRNA amplicon (V4 region) sequencing analysis |
| Zou et al. 2020(Zou et al., 2020) | China | 48 patients with ASD (5 [2-7] years old, 17.4 [13.7 – 26.5] kg/m², 10 female)  48 control (4 years old, 16.3 [13.9 – 21.3] kg/m², 24 female). | 16S rRNA amplicon (V3-V4 regions) sequencing analysis |
| Ahmed et al. 2020(Ahmed et al., 2020) | Egypt | 41 patients with ASD (5.5 ± 2 years old, BMI unknown, 13 female)  45 control (5.3 ± 3 years old, BMI unknown, 7 female). | 16S rRNA amplicon (V3-V5 regions) sequencing analysis |

Adams, J. B., Johansen, L. J., Powell, L. D., Quig, D., and Rubin, R. A. (2011). Gastrointestinal flora and gastrointestinal status in children with autism--comparisons to typical children and correlation with autism severity. *BMC Gastroenterol.* 11, 22. doi:10.1186/1471-230X-11-22.

Ahmed, S. A., Elhefnawy, A. M., Azouz, H. G., Roshdy, Y. S., Ashry, M. H., Ibrahim, A. E., et al. (2020). Study of the gut Microbiome Profile in Children with Autism Spectrum Disorder: a Single Tertiary Hospital Experience. *J. Mol. Neurosci. MN* 70, 887–896. doi:10.1007/s12031-020-01500-3.

Chen, Y., Fang, H., Li, C., Wu, G., Xu, T., Yang, X., et al. (2020). Gut Bacteria Shared by Children and Their Mothers Associate with Developmental Level and Social Deficits in Autism Spectrum Disorder. *mSphere* 5. doi:10.1128/mSphere.01044-20.

Ding, X., Xu, Y., Zhang, X., Zhang, L., Duan, G., Song, C., et al. (2020). Gut microbiota changes in patients with autism spectrum disorders. *J. Psychiatr. Res.* 129, 149–159. doi:10.1016/j.jpsychires.2020.06.032.

Finegold, S. M., Summanen, P. H., Downes, J., Corbett, K., and Komoriya, T. (2017). Detection of Clostridium perfringens toxin genes in the gut microbiota of autistic children. *Anaerobe* 45, 133–137. doi:10.1016/j.anaerobe.2017.02.008.

Gondalia, S. V., Palombo, E. A., Knowles, S. R., Cox, S. B., Meyer, D., and Austin, D. W. (2012). Molecular characterisation of gastrointestinal microbiota of children with autism (with and without gastrointestinal dysfunction) and their neurotypical siblings. *Autism Res. Off. J. Int. Soc. Autism Res.* 5, 419–427. doi:10.1002/aur.1253.

Iovene, M. R., Bombace, F., Maresca, R., Sapone, A., Iardino, P., Picardi, A., et al. (2017). Intestinal Dysbiosis and Yeast Isolation in Stool of Subjects with Autism Spectrum Disorders. *Mycopathologia* 182, 349–363. doi:10.1007/s11046-016-0068-6.

Kang, D.-W., Ilhan, Z. E., Isern, N. G., Hoyt, D. W., Howsmon, D. P., Shaffer, M., et al. (2018). Differences in fecal microbial metabolites and microbiota of children with autism spectrum disorders. *Anaerobe* 49, 121–131. doi:10.1016/j.anaerobe.2017.12.007.

Kang, D.-W., Park, J. G., Ilhan, Z. E., Wallstrom, G., Labaer, J., Adams, J. B., et al. (2013). Reduced incidence of Prevotella and other fermenters in intestinal microflora of autistic children. *PloS One* 8, e68322. doi:10.1371/journal.pone.0068322.

Kushak, R. I., Winter, H. S., Buie, T. M., Cox, S. B., Phillips, C. D., and Ward, N. L. (2017). Analysis of the Duodenal Microbiome in Autistic Individuals: Association With Carbohydrate Digestion. *J. Pediatr. Gastroenterol. Nutr.* 64, e110–e116. doi:10.1097/MPG.0000000000001458.

Liu, S., Li, E., Sun, Z., Fu, D., Duan, G., Jiang, M., et al. (2019). Altered gut microbiota and short chain fatty acids in Chinese children with autism spectrum disorder. *Sci. Rep.* 9, 287. doi:10.1038/s41598-018-36430-z.

Luna, R. A., Oezguen, N., Balderas, M., Venkatachalam, A., Runge, J. K., Versalovic, J., et al. (2016). Distinct Microbiome-Neuroimmune Signatures Correlate With Functional Abdominal Pain in Children With Autism Spectrum Disorder. *Cell. Mol. Gastroenterol. Hepatol.* 3, 218–230. doi:10.1016/j.jcmgh.2016.11.008.

Ma, B., Liang, J., Dai, M., Wang, J., Luo, J., Zhang, Z., et al. (2019). Altered Gut Microbiota in Chinese Children With Autism Spectrum Disorders. *Front. Cell. Infect. Microbiol.* 9, 40. doi:10.3389/fcimb.2019.00040.

Plaza-Díaz, J., Gómez-Fernández, A., Chueca, N., de la Torre-Aguilar, M. J., Gil, Á., Perez-Navero, J. L., et al. (2019). Autism Spectrum Disorder (ASD) with and without Mental Regression Is Associated with Changes in the Fecal Microbiota. *Nutrients* 11. doi:10.3390/nu11020337.

Pulikkan, J., Maji, A., Dhakan, D. B., Saxena, R., Mohan, B., Anto, M. M., et al. (2018). Gut Microbial Dysbiosis in Indian Children with Autism Spectrum Disorders. *Microb. Ecol.* 76, 1102–1114. doi:10.1007/s00248-018-1176-2.

Rose, D. R., Yang, H., Serena, G., Sturgeon, C., Ma, B., Careaga, M., et al. (2018). Differential immune responses and microbiota profiles in children with autism spectrum disorders and co-morbid gastrointestinal symptoms. *Brain. Behav. Immun.* 70, 354–368. doi:10.1016/j.bbi.2018.03.025.

Son, J. S., Zheng, L. J., Rowehl, L. M., Tian, X., Zhang, Y., Zhu, W., et al. (2015). Comparison of Fecal Microbiota in Children with Autism Spectrum Disorders and Neurotypical Siblings in the Simons Simplex Collection. *PloS One* 10, e0137725. doi:10.1371/journal.pone.0137725.

Strati, F., Cavalieri, D., Albanese, D., De Felice, C., Donati, C., Hayek, J., et al. (2017). New evidences on the altered gut microbiota in autism spectrum disorders. *Microbiome* 5. doi:10.1186/s40168-017-0242-1.

Sun, H., You, Z., Jia, L., and Wang, F. (2019). Autism spectrum disorder is associated with gut microbiota disorder in children. *BMC Pediatr.* 19, 516. doi:10.1186/s12887-019-1896-6.

Tomova, A., Husarova, V., Lakatosova, S., Bakos, J., Vlkova, B., Babinska, K., et al. (2015). Gastrointestinal microbiota in children with autism in Slovakia. *Physiol. Behav.* 138, 179–187. doi:10.1016/j.physbeh.2014.10.033.

Wang, L., Christophersen, C. T., Sorich, M. J., Gerber, J. P., Angley, M. T., and Conlon, M. A. (2011). Low relative abundances of the mucolytic bacterium Akkermansia muciniphila and Bifidobacterium spp. in feces of children with autism. *Appl. Environ. Microbiol.* 77, 6718–6721. doi:10.1128/AEM.05212-11.

Wang, L., Christophersen, C. T., Sorich, M. J., Gerber, J. P., Angley, M. T., and Conlon, M. A. (2013). Increased abundance of Sutterella spp. and Ruminococcus torques in feces of children with autism spectrum disorder. *Mol. Autism* 4, 42. doi:10.1186/2040-2392-4-42.

Zhai, Q., Cen, S., Jiang, J., Zhao, J., Zhang, H., and Chen, W. (2019). Disturbance of trace element and gut microbiota profiles as indicators of autism spectrum disorder: A pilot study of Chinese children. *Environ. Res.* 171, 501–509. doi:10.1016/j.envres.2019.01.060.

Zhang, M., Ma, W., Zhang, J., He, Y., and Wang, J. (2018). Analysis of gut microbiota profiles and microbe-disease associations in children with autism spectrum disorders in China. *Sci. Rep.* 8. doi:10.1038/s41598-018-32219-2.

Zou, R., Xu, F., Wang, Y., Duan, M., Guo, M., Zhang, Q., et al. (2020). Changes in the Gut Microbiota of Children with Autism Spectrum Disorder. *Autism Res.* 13, 1614–1625. doi:10.1002/aur.2358.
