# Supplementary material for: The Role of Gut Microbiota in Neuropsychiatric Diseases – Creation of An Atlas-Based on Quantified Evidence
Source: Front Cell Infect Microbiol. 2022 Mar 14;12:831666. doi: 10.3389/fcimb.2022.831666 (PMC8964285; doi:10.3389/fcimb.2022.831666)
Supplement: Supplementary file 2 [file Table_2.docx]

**Supplementary Table 2**: Characteristics of the included studies on Major Depressive Disorders (MDD).

| Study | Country | Participants | Microbiota analysis method |
| --- | --- | --- | --- |
| Naseribafrouei et al. 2014(Naseribafrouei et al., 2014) | Norway | 37 MDD patients (49.2 ± 14 years old, 25.2 ± 4kg/m²,19 female)  18 control (46.1 ± 14 years old, 24.7 ± 3 kg/m², 10 female). | 16S rRNA amplicon (V4 regions) sequencing analysis |
| Jiang et al. 2015(Jiang et al., 2015) | China | 29 MDD patients (25.3 ± 5 years old, 20.3 ± 3kg/m²,10 female)  30 control (27.1 ± 5 years old, 19.6 ± 3 kg/m², 15 female). | 16S rRNA amplicon (V4 regions) sequencing analysis |
| Aizawa et al. 2016(Aizawa et al., 2016) | Japan | 43 MDD patients (39.4 ± 10 years old, 23.2 ± 4kg/m²,20 female)  57 control (42.8 ± 13 years old, 22.3 ± 4 kg/m², 35female). | 16S rRNA amplicon (V3-V4 regions) sequencing analysis |
| Zheng et al. 2016(Zheng et al., 2016) | China | 58 MDD patients (40.6 ± 12 years old, 22.0 ± 3kg/m²,36 female)  63 control (41.8 ± 12 years old, 22.6 ± 3 kg/m², 40 female). | 16S rRNA amplicon (V4 regions) sequencing analysis |
| Kelly et al. 2016(Kelly et al., 2019) | Ireland | 34 MDD patients (41.8 ± 12 years old, 26.2 ± 4 kg/m², 13 female)  34 control (45.8 ± 12 years old, 24.6 ± 3kg/m², 14 female). | 16S rRNA amplicon (V3-V4 regions) sequencing analysis |
| Liu et al. 2016(Liu et al., 2016) | China | 15 MDD patients (44.8 ± 15 years old, 22.0 ± 3 kg/m², 11 female)  20 control (43.9 ± 11 years old, 24.6 ± 3kg/m², 13 female). | 16S rRNA amplicon (V1-V3 regions) sequencing analysis |
| Lin et al. 2017(Lin et al., 2017) | China | 10 MDD patients (36.2 ± 10 years old, 23.8 ± 2kg/m²,4 female)  10 control (38.1 ± 3 years old, 24.2 ± 2 kg/m², 4 female). | 16S rRNA amplicon (V4 regions) sequencing analysis |
| Chen et al. 2018(Chen et al., 2018b) | China | 10 MDD patients (43.9 ± 14 years old, 23.5 ± 2kg/m², 5 female)  10 control (39.6 ± 9 years old, 22.6 ± 2 kg/m², 5 female). | 16S rRNA amplicon (V4 regions) sequencing analysis |
| Valles-Colomer et al. 2019(Valles-Colomer et al., 2019) | Belgium | 121 MDD patients  933 controls (50 years old, 24.9 kg/m²) | 16S rRNA amplicon (V4 regions) sequencing analysis |
| Lai et al. 2019(Lai et al., 2019) | China | 26 MDD patients (43.7 ± 11 years old, 21.1 ± 2kg/m², 18 female)  29 control (39.4 ± 11 years old, 21.1 ± 2 kg/m², 16 female). | shotgun metagenomic sequencing |
| Chen et al. 2019(Chen et al., 2018a) | China | 44 MDD patients (41.7 ± 11 years old, 22.2 ± 2kg/m², 24 female)  44 control (39.4 ± 11 years old, 22.1 ± 2 kg/m², 24 female). | 16S rRNA amplicon (V3-V5 regions) sequencing analysis |
| Chung et al. 2019(Chung et al., 2019) | China | 36 MDD patients (45.8 ± 14 years old, 22.8 ± 4kg/m², 28 female)  37 control (41.2 ± 13 years old, 23.9 ± 4 kg/m², 23 female). | 16S rRNA amplicon (V4 regions) sequencing analysis |
| Rong et al. 2019(Rong et al., 2019) | China | 31 MDD patients (41.6 ± 10 years old, 21.4 ± 2kg/m², 22 female)  30 control (49.5 ± 10 years old, 21.9 ± 3 kg/m², 14 female). | Shotgun metagenomic sequencing |
| Mason et al. 2020(Mason et al., 2020) | USA | 38 MDD patients (39.2 ± 12 years old, 22.8 ± 4kg/m², 31 female)  10 control (33.1 ± 8 years old, 25.6 ± 4 kg/m², 23 female). | 16S rRNA amplicon (V4 regions) sequencing analysis |
| Fontana et al. 2020(Fontana et al., 2020) | Italy | 34 MDD patients (57 [43 - 61] years old, 23.1 [21.9 – 28.1] kg/m², 24 female)  20 control (37.7 [30.6 – 58.0] years old, 22.7 [21.1 – 23.8] kg/m², 7 female). | 16S rRNA amplicon (V3-V4 regions) sequencing analysis |
| Liu et al. 2020(Liu et al., 2020) | USA | 43 MDD patients (21.9 ± 2 years old, BMI unknown, 38 female)  47 control (22.1 ± 2 years old, BMI unknown, 34 female). | 16S rRNA amplicon (V4 regions) sequencing analysis |
| Chen et al. 2020(Chen et al., 2020) | China | 70 MDD patients (39.2 ± 12 years old, 22.8 ± 4kg/m², 31 female)  71 control (33.1 ± 8 years old, 25.6 ± 4 kg/m², 23 female). | 16S rRNA amplicon (V4 regions) sequencing analysis |
| Zheng et al. 2020(Zheng et al., 2020) | China | 122 MDD patients (26.5 ± 4 years old, 22.4 ± 4 kg/m², 78 female)  171 control (26.8 ± 6 years old, 22.1 ± 3kg/m², 101 female). | 16S rRNA amplicon (V4 regions) sequencing analysis |

Aizawa, E., Tsuji, H., Asahara, T., Takahashi, T., Teraishi, T., Yoshida, S., et al. (2016). Possible association of Bifidobacterium and Lactobacillus in the gut microbiota of patients with major depressive disorder. *J. Affect. Disord.* 202, 254–257. doi:10.1016/j.jad.2016.05.038.

Chen, J.-J., He, S., Fang, L., Wang, B., Bai, S.-J., Xie, J., et al. (2020). Age-specific differential changes on gut microbiota composition in patients with major depressive disorder. *Aging* 12, 2764–2776. doi:10.18632/aging.102775.

Chen, J.-J., Zheng, P., Liu, Y.-Y., Zhong, X.-G., Wang, H.-Y., Guo, Y.-J., et al. (2018a). Sex differences in gut microbiota in patients with major depressive disorder. *Neuropsychiatr. Dis. Treat.* 14, 647–655. doi:10.2147/NDT.S159322.

Chen, Z., Li, J., Gui, S., Zhou, C., Chen, J., Yang, C., et al. (2018b). Comparative metaproteomics analysis shows altered fecal microbiota signatures in patients with major depressive disorder. *Neuroreport* 29, 417–425. doi:10.1097/WNR.0000000000000985.

Chung, Y.-C. E., Chen, H.-C., Chou, H.-C. L., Chen, I.-M., Lee, M.-S., Chuang, L.-C., et al. (2019). Exploration of microbiota targets for major depressive disorder and mood related traits. *J. Psychiatr. Res.* 111, 74–82. doi:10.1016/j.jpsychires.2019.01.016.

Fontana, A., Manchia, M., Panebianco, C., Paribello, P., Arzedi, C., Cossu, E., et al. (2020). Exploring the Role of Gut Microbiota in Major Depressive Disorder and in Treatment Resistance to Antidepressants. *Biomedicines* 8. doi:10.3390/biomedicines8090311.

Jiang, H., Ling, Z., Zhang, Y., Mao, H., Ma, Z., Yin, Y., et al. (2015). Altered fecal microbiota composition in patients with major depressive disorder. *Brain. Behav. Immun.* 48, 186–194. doi:10.1016/j.bbi.2015.03.016.

Kelly, J. R., Keane, V. O., Cryan, J. F., Clarke, G., and Dinan, T. G. (2019). Mood and Microbes: Gut to Brain Communication in Depression. *Gastroenterol. Clin. North Am.* 48, 389–405. doi:10.1016/j.gtc.2019.04.006.

Lai, W., Deng, W., Xu, S., Zhao, J., Xu, D., Liu, Y., et al. (2019). Shotgun metagenomics reveals both taxonomic and tryptophan pathway differences of gut microbiota in major depressive disorder patients. *Psychol. Med.*, 1–12. doi:10.1017/S0033291719003027.

Lin, P., Ding, B., Feng, C., Yin, S., Zhang, T., Qi, X., et al. (2017). Prevotella and Klebsiella proportions in fecal microbial communities are potential characteristic parameters for patients with major depressive disorder. *J. Affect. Disord.* 207, 300–304. doi:10.1016/j.jad.2016.09.051.

Liu, R. T., Rowan-Nash, A. D., Sheehan, A. E., Walsh, R. F. L., Sanzari, C. M., Korry, B. J., et al. (2020). Reductions in anti-inflammatory gut bacteria are associated with depression in a sample of young adults. *Brain. Behav. Immun.* 88, 308–324. doi:10.1016/j.bbi.2020.03.026.

Liu, Y., Zhang, L., Wang, X., Wang, Z., Zhang, J., Jiang, R., et al. (2016). Similar Fecal Microbiota Signatures in Patients With Diarrhea-Predominant Irritable Bowel Syndrome and Patients With Depression. *Clin. Gastroenterol. Hepatol.* 14, 1602-1611.e5. doi:10.1016/j.cgh.2016.05.033.

Mason, B. L., Li, Q., Minhajuddin, A., Czysz, A. H., Coughlin, L. A., Hussain, S. K., et al. (2020). Reduced anti-inflammatory gut microbiota are associated with depression and anhedonia. *J. Affect. Disord.* 266, 394–401. doi:10.1016/j.jad.2020.01.137.

Naseribafrouei, A., Hestad, K., Avershina, E., Sekelja, M., Linløkken, A., Wilson, R., et al. (2014). Correlation between the human fecal microbiota and depression. *Neurogastroenterol. Motil. Off. J. Eur. Gastrointest. Motil. Soc.* 26, 1155–1162. doi:10.1111/nmo.12378.

Rong, H., Xie, X., Zhao, J., Lai, W., Wang, M., Xu, D., et al. (2019). Similarly in depression, nuances of gut microbiota: Evidences from a shotgun metagenomics sequencing study on major depressive disorder versus bipolar disorder with current major depressive episode patients. *J. Psychiatr. Res.* 113, 90–99. doi:10.1016/j.jpsychires.2019.03.017.

Valles-Colomer, M., Falony, G., Darzi, Y., Tigchelaar, E. F., Wang, J., Tito, R. Y., et al. (2019). The neuroactive potential of the human gut microbiota in quality of life and depression. *Nat. Microbiol.* 4, 623–632. doi:10.1038/s41564-018-0337-x.

Zheng, P., Yang, J., Li, Y., Wu, J., Liang, W., Yin, B., et al. (2020). Gut Microbial Signatures Can Discriminate Unipolar from Bipolar Depression. *Adv. Sci. Weinh. Baden-Wurtt. Ger.* 7, 1902862. doi:10.1002/advs.201902862.

Zheng, P., Zeng, B., Zhou, C., Liu, M., Fang, Z., Xu, X., et al. (2016). Gut microbiome remodeling induces depressive-like behaviors through a pathway mediated by the host’s metabolism. *Mol. Psychiatry* 21, 786–796. doi:10.1038/mp.2016.44.
