# Supplementary material for: The Role of Gut Microbiota in Neuropsychiatric Diseases – Creation of An Atlas-Based on Quantified Evidence
Source: Front Cell Infect Microbiol. 2022 Mar 14;12:831666. doi: 10.3389/fcimb.2022.831666 (PMC8964285; doi:10.3389/fcimb.2022.831666)
Supplement: Supplementary file 3 [file Table_3.docx]

**Supplementary Table 3:** Characteristics of the included studies on Schizophrenia.

| Study | Country | Participants | Microbiota analysis method |
| --- | --- | --- | --- |
| Schwartz et al 2018(Schwarz et al., 2018) | Germany | 28 first episode schizophrenia patients (25.9 ± 5 years old, 23.5 ± 4 kg/m², 12 female)  16 control (27.1 ± 6 years old, 23.1 ± 3 kg/m², 8 female). | 16S rRNA amplicon (V3-V4 regions) sequencing analysis |
| Shen et al. 2018(Shen et al., 2018) | China | 64 Schizophrenia patients (42.0 ± 11 years old, 23.5 ± 4 kg/m², 28 female)  53 control (39.0 ± 14 years old, 23.1 ± 3 kg/m², 18 female). | 16S rRNA amplicon (V3-V4 regions) sequencing analysis |
| Xu et al. 2019(Xu et al., 2019) | China | 44 Schizophrenia patients (35.0 ± 11 years old, 22.0 ± 3 kg/m², 16 female)  44 control (35.0 ± 11 years old, 23.1 ± 3 kg/m², 16 female). | 16S rRNA amplicon (V4 regions) sequencing analysis |
| Nguyen et al. 2019(Nguyen et al., 2019) | USA | 25 Schizophrenia patients (52.9 ± 11 years old, 31.8 ± 5 kg/m², 11 female)  25 control (54.7 ± 11 years old, 28.9 ± 4 kg/m², 10 female). | 16S rRNA amplicon (V4 regions) sequencing analysis |
| Li et al. 2020(Li et al., 2020) | China | 29 Schizophrenia patients (41.0 ± 14 years old, 23.0 ± 3 kg/m², 41 female)  82 control (42.1 ± 13 years old, 24.5 ± 4 kg/m², 36 female). | 16S rRNA amplicon (V4 regions) sequencing analysis |
| Pan et al. 2020(Pan et al., 2020) | China | 29 Schizophrenia patients (34.9 ± 11 years old, 23.7 ± 3 kg/m², 19 female)  29 control (34.8 ± 11 years old, 23.5 ± 2 kg/m², 19 female). | 16S rRNA amplicon (V3-V4 regions) sequencing analysis |
| Ma et al. 2020(Ma et al., 2020) | China | 40 Schizophrenia patients (34.9 ± 11 years old, 23.7 ± 3 kg/m², 19 female)  69 control (34.8 ± 11 years old, 23.5 ± 2 kg/m², 19 female). | 16S rRNA amplicon (V4 regions) sequencing analysis |
| Zhang et al. 2020(Zhang et al., 2020) | China | 10 Schizophrenia patients (37.6 ± 7 years old, 23.3 ± 3 kg/m², 4 female)  16 control (35.8 ± 7 years old, 22.3 ± 6 kg/m², 7 female). | 16S rRNA amplicon (V4 regions) sequencing analysis |
| Xu et al. 2020(Xu et al., 2020) | China | 84 Schizophrenia patients (35.0 ± 11 years old, 22.0 ± 3 kg/m², 36 female)  84 control (35.0 ± 11 years old, 23.1 ± 3 kg/m², 36 female). | 16S rRNA amplicon (V4 regions) sequencing analysis |

Li, S., Zhuo, M., Huang, X., Huang, Y., Zhou, J., Xiong, D., et al. (2020). Altered gut microbiota associated with symptom severity in schizophrenia. *PeerJ* 8, e9574. doi:10.7717/peerj.9574.

Ma, X., Asif, H., Dai, L., He, Y., Zheng, W., Wang, D., et al. (2020). Alteration of the gut microbiome in first-episode drug-naïve and chronic medicated schizophrenia correlate with regional brain volumes. *J. Psychiatr. Res.* 123, 136–144. doi:10.1016/j.jpsychires.2020.02.005.

Nguyen, T. T., Kosciolek, T., Maldonado, Y., Daly, R. E., Martin, A. S., McDonald, D., et al. (2019). Differences in gut microbiome composition between persons with chronic schizophrenia and healthy comparison subjects. *Schizophr. Res.* 204, 23–29. doi:10.1016/j.schres.2018.09.014.

Pan, R., Zhang, X., Gao, J., Yi, W., Wei, Q., and Su, H. (2020). Analysis of the diversity of intestinal microbiome and its potential value as a biomarker in patients with schizophrenia: A cohort study. *Psychiatry Res.* 291, 113260. doi:10.1016/j.psychres.2020.113260.

Schwarz, E., Maukonen, J., Hyytiäinen, T., Kieseppä, T., Orešič, M., Sabunciyan, S., et al. (2018). Analysis of microbiota in first episode psychosis identifies preliminary associations with symptom severity and treatment response. *Schizophr. Res.* 192, 398–403. doi:10.1016/j.schres.2017.04.017.

Shen, Y., Xu, J., Li, Z., Huang, Y., Yuan, Y., Wang, J., et al. (2018). Analysis of gut microbiota diversity and auxiliary diagnosis as a biomarker in patients with schizophrenia: A cross-sectional study. *Schizophr. Res.* 197, 470–477. doi:10.1016/j.schres.2018.01.002.

Xu, R., Wu, B., Liang, J., He, F., Gu, W., Li, K., et al. (2019). Altered gut microbiota and mucosal immunity in patients with schizophrenia. *Brain. Behav. Immun.* doi:10.1016/j.bbi.2019.06.039.

Xu, R., Wu, B., Liang, J., He, F., Gu, W., Li, K., et al. (2020). Altered gut microbiota and mucosal immunity in patients with schizophrenia. *Brain. Behav. Immun.* 85, 120–127. doi:10.1016/j.bbi.2019.06.039.

Zhang, X., Pan, L.-Y., Zhang, Z., Zhou, Y.-Y., Jiang, H.-Y., and Ruan, B. (2020). Analysis of gut mycobiota in first-episode, drug-naïve Chinese patients with schizophrenia: A pilot study. *Behav. Brain Res.* 379, 112374. doi:10.1016/j.bbr.2019.112374.
