# Supplementary material for: The Role of Gut Microbiota in Neuropsychiatric Diseases – Creation of An Atlas-Based on Quantified Evidence
Source: Front Cell Infect Microbiol. 2022 Mar 14;12:831666. doi: 10.3389/fcimb.2022.831666 (PMC8964285; doi:10.3389/fcimb.2022.831666)
Supplement: Supplementary file 5 [file Table_5.docx]

**Supplementary Table 5:** Individual results of the included studies of MDD. For the individual studies, red color indicates an increased of the relative abundance, blue a decrease of relative abundance. At the taxa level, the green color indicates the replicated metabolites, orange colors indicates metabolites found in opposite directions.
